# Provably lightweight and secure IoHT scheme with post-quantum cryptography and fog computing: A comprehensive scheme for healthcare system

**DOI:** 10.1016/j.mex.2025.103631

**Published:** 2025-09-18

**Authors:** Enas W. Abood, Ali A. Yassin, Zaid Ameen Abduljabbar, Vincent Omollo Nyangaresi, Ali Hasan Ali

**Affiliations:** a Department of Mathematics, College of Science, University of Basrah, Basrah 61004, Iraq; b Department of Computer Science, College of Education for Pure Sciences, University of Basrah, Basrah 61004, Iraq; c Department of Business Management, Al-Imam University College, Balad 34011, Iraq; d Shenzhen Institute, Huazhong University of Science and Technology, Shenzhen 518000, China; e Department of Computer Science and Software Engineering, Jaramogi Oginga Odinga University of Science and Technology, Bondo 40601, Kenya; f Department of Applied Electronics, Saveetha School of Engineering, SIMATS, Chennai, Tamil Nadu 602105, India; g Department of Mathematics, College of Education for Pure Sciences, University of Basrah, Basrah 61004, Iraq; h Technical Engineering College, Al-Ayen University, Thi-Qar 64001, Iraq; i Institute of Mathematics, University of Debrecen, Pf. 400, Debrecen H4002, Hungary

**Keywords:** IoHT, Post-quantum cryptography, Key encapsulation mechanism (KEM), Role-based access control (RBAC), Symmetric searchable encryption (SSE)

## Abstract

Quantum computers threaten the security of commonly used public-key cryptosystems, such as Rivest-Shamir-Adleman (RSA) and Elliptic Curve Cryptography (ECC). This is because these quantum computers efficiently solve factorization and discrete logarithm problems, which are the basis for RSA and ECC. This compromises confidentiality, integrity, and authenticity in security systems deployed in various applications, such as Internet-of-Healthcare Things (IoHT). Post-quantum cryptography, while a potential solution, introduces computational overheads that can slow service delivery. This poses serious concerns in IoHT safety critical systems as it directly impacts patient safety and healthcare systems. This paper aims to design a post-quantum resistance scheme for IoHTs. Our scheme is based on ECC, Counter mode (CTR), and Key Encapsulation Mechanism (KEM). To guarantee the safety and storage of electronic records for the network entities and support scalability, blockchain and InterPlanetary File System (IPFS) were employed. To achieve improved levels of security and more effective control when accessing specific data and resources, we apply Role-Based Access Control (RBAC). In addition, we deploy Symmetric Searchable Encryption (SSE) for efficient and secure data search. The scheme's security was formally verified using the Scyther tool, and Burrows–Abadi–Needham (BAN) logic. In addition, informal security analysis shows that our proposed scheme offers mutual authentication, confidentiality, integrity, and other security requirements. In addition, it withstands well-known threats and some of the recent threats, such as phishing, quantum, and 51% attacks. Moreover, a comparative analysis was conducted with other related protocols to show the efficiency of the proposed scheme in the IoHT environment. The results indicate that the computation overhead was reduced by 90%, while communication cost and latency were relatively low. On the other hand, throughput was greatly increased while energy consumption was very low.

The proposed scheme is a low complexity solution for IoHT environments to address existing threats and maintain data integrity.

Blockchain and IPFS ensure secure, scalable e-record storage for network entities.

Achieving secure, effective access control with RBAC and enhancing data searchability with SSE.

## Graphical abstract

**Figure:**
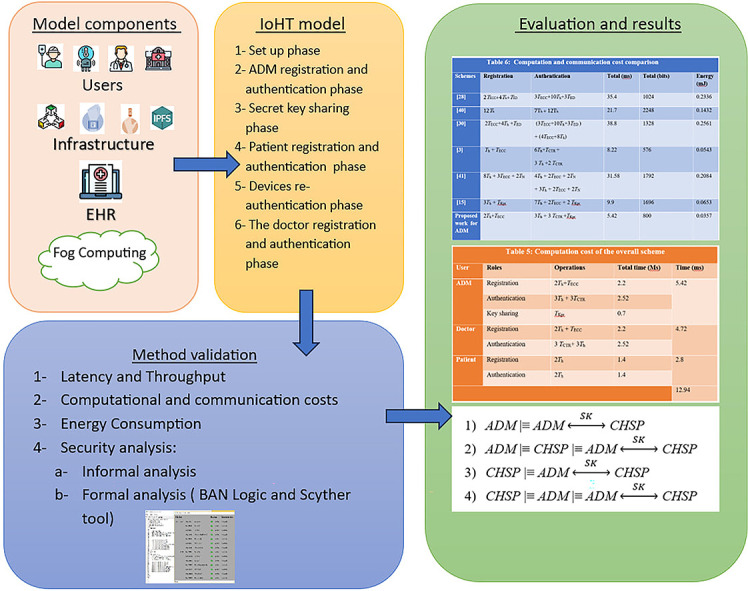

Specifications table

**Table:** 

**Subject area**	Computer Science
**More specific subject area**	**Internet of Health Thing (IoHT)**
**Name of your method**	**lightweight and Secure Authentication IoHT Scheme**
**Name and reference of original method**	**I have adapted the methods from the following key references:** **Kyper algorithm :** **C. Rubio García et al., “Quantum-resistant Transport Layer Security,” Comput Commun, vol. 213, pp. 345–358, Jan. 2024, doi: 10.1016/j.comcom.2023.11.010.** **ECC and KEM methods :** **A. Braeken, “Flexible hybrid post-quantum bidirectional multi-factor authentication and key agreement framework using ECC and KEM,” Future Generation Computer Systems, vol. 166, May 2025, doi: 10.1016/j.future.2024.107634**
**Resource availability**	**All provided resources are publicly accessible. Details are described in the (Basics of deployed techniques) Section 3. for reproducibility.** **Equipment and hardware: ThinkPad Lenovo (13th Gen Intel(R) Core(TM) i7–13700H 2.40GHz).** **Software: Improved codes with Python from GitHub.**

## Introduction

The IoHT has become one of the most important technological developments in the healthcare sector [1]. This environment is characterized with the integration of medical devices, telemedicine, daily health monitoring systems and programs embedded in smart devices and personal phones [2]. As explained in [3,4], there has been wide deployment and utilization of IoHT among the healthcare providers. In spite of this, the IoHT environment is susceptible to numerous security threats. These vulnerabilities and security threats revolve around data privacy, authentication, access control, cyber-attacks and threats. As such, strengthening systems against attacks is essential to maintain IoHT security and privacy [5]. The security requirements in IoHT include confidentiality, non-repudiation, data integrity, as well as the authentication of devices and users [6]. Authentication is crucial for meeting these security requirements as it helps confirm the identity of entities interacting within the system [7]. However, most of the current authentication and authorization schemes are vulnerable to attacks due to the multiplicity of devices and users within the network [8]. As such, strong authentication schemes must be developed to mitigate these attacks [9]. In line with this, many studies and methods have emerged that can contribute to building and developing strong systems. These current systems integrate revolutionary blockchain (BC) technology with techniques such as multi-factor authentication [10], biometric authentication [11], mutual authentication [12], as well as digital certificates and tokens [13,14]. However, most of these techniques face challenges such as vulnerability to quantum attacks, high costs, and applicability.

Over the recent past, there has been continuous progress in the development of both offensive and defensive cyber security tools. These techniques are specifically designed for launching attacks, decrypting data. In addition, the emerging quantum computers with their high computational potential pose significant challenges in securing data exchange due to their capabilities of breaking previously secure cryptographic codes. These quantum attacks can break the widely used public key encryption algorithms such as ECC, Counter mode encryption (CTR) and RSA. These algorithms are hinged on the difficulties of solving discrete logarithms and factorizations of large numbers [15]. To confront such attacks, there is need for the development of robust protection tools and encryption methods [16]. As such, researchers have begun to develop secure alternatives through Key Encapsulation Mechanisms (KEM) and signature encapsulation such as CRYSTALS-Kyber. Kyber, a NIST post-quantum finalist KEM, relies on the hardness of solving module-LWE. It provides three parameter sets: Kyber-512 (AES-128 equivalent), Kyber-768 (AES-192 equivalent), and Kyber-1024 (AES-256 equivalent).

In the proposed scheme, we deploy kyper-512 to achieve a balance between computation and security performance in real-time applications [17]. In most of the IoHT studies, centralized and decentralized authentication methods are predominantly described. In these studies, decentralized approaches using BC technology are increasingly recommended due to their suitability for the distributed nature of IoHT systems [18,19]. The studies described some of the blockchain’s fundamental features such as consensus, immutability, decentralization, and security. These characteristics enable BC to improve data authentication and management, maintain data trustworthiness, enhance data sharing, increase privacy and security, and boost the quality of big data [19,20]. Another decentralized tool is InterPlanetary File System (IPFS), which is characterized by high performance during storage and retrieval of large amounts of data, as well as quick access to stored files. According to [2,21], IPFS provides high levels of security. However, unlike BC, the stored data is not encrypted by default. Therefore, supplemental encryption algorithm must be deployed to protect sensitive data. In addition, special measures must be taken to ensure privacy, based on the storage settings [3,22]. Due to its ability to handle large files, IPFS is suitable for large medical files, which require fast retrieval at lower costs when compared with BC [23]. To achieve both speed and security, our proposed scheme combines both techniques: using IPFS to store large medical files, and BC to store the hashes of these files [24].

Among the feasible solutions for data security and storage is SSE that handles secure searches. However, it faces challenges when dealing with large databases due to poor performance and locality [56]. Enhancements such as inverted index have been put forward to provide security while at the same time facilitating fast storage and retrieval. This helps increase storage capacity and read efficiency [57]. For privacy preservation and data security, RBAC is deployed, where it helps define, assign authority, as well as regulates access and use of data [2]. This paper makes the following contributions, which are geared towards designing a secure and low complexity mutual authentication scheme for IoHT:

• A new post-quantum scheme is developed to meet all critical security requirements and resist numerous types of well-known cyber and quantum attacks with low computational costs and high performance.
• The proposed scheme employs ECC and CTR for securing data transmission during authentication, and the Kyper-512 encapsulation method to secure shared key distribution.
• The scheme leverages fog computing with BC and IPFS to provide a trustable, feasible, and efficient storage decentralized environment while ensuring high scalability.
• The proposed scheme enforces RBAC for regulating data access and managing users (doctors, patients, medical staff, and medical devices) through a central health provider and administrator. In addition, it utilizes SSE for more reliable and efficient search mechanism from the device list. It also supports secure, low-cost device reconnection.
• The scheme's efficiency has been formally and informally analyzed and proofed. We implemented a formal security analysis using Scyther, with results demonstrating the scheme's resilience against known threats. Additionally, we employed BAN logic as formal proof of its security functionalities.
• The performance evaluation has proven the scheme’s efficiency, which has then been compared to similar works in terms of computational, communication costs and energy.

The rest of this paper is structured as follows: A review of related works and analysis of existing IoHT schemes are given in "Related works", while "Basics of deployed techniques" describes primitive cryptographic operations deployed in this work. In "Security goals and threats", the security requirements and threats are outlined, while the proposed quantum authentication scheme using ECC and KEM is presented in "Proposed scheme details". On the other hand, the performance analysis and associated costs are discussed in "Method validation", while "Conclusion" presents the formal and informal security analyses using BAN logic and Scyther. Finally, Section 8 concludes the paper and provides some future research scopes.

## Related works

The rapid advances in IoHT systems and technologies have brought forth numerous privacy and security challenges during data exchange and storage. Therefore, many researchers have developed different authentication protocols for privacy preservation and threat mitigation. These authentication schemes use different cryptographic operations and network architectures. For instance, some protocols are based on cloud servers, encryption, multi-factor, and biometric authentication. In addition, they prevent various external and internal attacks, and deploy diverse analysis methods. The following sub-sections discuss the most recent developments in this domain.

### Pre-quantum cryptography

To secure EHR systems, Feng et al. [25] proposed an authentication system based on homomorphic encryption that enhances security with high performance. It is a secure two-party authentication protocol that exchanges the original private key, and ensures that only authorized parties have access to the system. However, it is not resistant to Distributed Denial of Service (DDoS) and brute-force attacks. In addition, it is unsuitable for mobile devices due to its high computation costs. On the other hand, Oliveira et al. [26] have proposed a security protocol to regulate access to encrypted electronic medical records (EMR) in emergency cases. This protocol is based on hybrid encryption approach consisting of Dynamic index-based Symmetric Searchable Encryption (DSSE) and Attribute-Based Encryption (ABE). It improves real-time access during acute care through enhanced speed and lower complexity in its operations. However, its resilience against cyberattacks such as Man-In-the-Middle (MIMT), DDoS, and spoofing remains unproven.

Yadav et al. [27] have introduced a lightweight dual-factor authentication protocol based on Extensible Authentication Protocol (EAP) encryption algorithms for securing Wireless Local Area Network (WLAN) connected IoT. The proposed protocol utilized random numbers, hash functions, and a combination of symmetric-key, public-key, and hash-based cryptography. Although the protocol enhanced data security, it cannot withstand DDoS attacks. On the other hand, Das et al. [28] presented a system that uses the Ciphertext Policy Attribute-Based Encryption (CP-ABE) technique based on ECC to manage access control for data and resources. Furthermore, they utilized multiple attributes to manage key generation. To reduce computational burden on the end users during decryption, cloud servers are deployed. This system is resistant to forgery, collusion, and MITM attacks. However, it cannot mitigate compromises to data security by external entities after decryption. To provide security services for cloud healthcare system end-users in IoHTs, Abbasi et al. [30] have introduced an ECC-based lightweight and robust authentication scheme based on fuzzy extractor method. However, it has not been proven against spoofing attacks.

### Post-quantum cryptography

Rubio et al. [17] have implemented two hybrid Quantum Key Distribution (QKD) and Post-Quantum Cryptography (PQC) based authenticated key exchange solutions based on concatenation and XOR operations. The proposed solutions enhanced handshake security with a performance cost in both time and complexity of approximately 117 % during the key establishment compared to classical methods. On the other hand, Wang et al. [31] proposed a lightweight post-quantum access and authentication scheme relying on lattice cryptography for satellite networks. They utilized hash function based on the Shortest Vector Problem (SVP) to maintain confidentiality, and a bonsai tree algorithm for time minimization. However, this scheme has not been proven against cyber-attacks.

Based on ECC and KEM, Braeken [15] has proposed a flexible hybrid authentication and key agreement framework for a client-server architecture. In addition, multi-factor authentication is also implemented using biometric authentication on the user side, and Physically Unclonable Functions (PUFs) on the device side. However, this framework incurs high communication and storage costs. In addition, it is vulnerable to physical layer attacks, and tends to be expensive due to the complexity and costs associated with PUFs. Therefore, Xu et al. [16] have introduced a Lattice-Based Forward-Secure Blind Signature (LFSBS) scheme for medical privacy preservation. This scheme offers forward security for data using a binary tree structure. In addition, it uses Small Integer Solution (SIS) to mitigate quantum attacks. Moreover, it can mitigate forgery and incurs low computational costs.

### Decentralized tools

Many researchers have adopted blockchain technology to develop secure and robust authentication schemes to protect healthcare data. For instance, Jaiman et al. [32] have introduced a consent model based on the Ethereum blockchain for health data-sharing platforms. By using Data Use Ontology (DUO) and Automatable Discovery and Access Matrix (ADA-M), their technique ensured secure data access. In addition, smart contracts are deployed to manage data access requests and enhance accountability, flexibility, and personalization while ensuring compliance with GDPR. However, scalability and data privacy were neglected. On the other hand, Jeong et al. [33] have applied blockchain technology to enhance the reliability of personal information management in intelligent healthcare. The data was collected through various measurement sensors. Thereafter, a system was designed to analyze biometric information such as electrocardiogram (ECG), leveraging on these sensors. Nonetheless, this system can easily compromise data integrity [34].

Azbeg et al. [2] and Alnuaimi et al. [35] have proposed healthcare models based on private Ethereum that utilized off-chain storage IPFS for data storage. These models use smart contracts to maintain traceability and trustworthiness. Unfortunately, these systems are unable to withstand some attacks such as DoS, cannot support privacy and scalability, and incur high complexities [80]. Similarly, Agha [36] proposed the application of blockchain technology and its integration with sensors to monitor patients in real-time. In addition, this solution maintains medical records to preserve patient privacy in a decentralized framework. However, this system has limited scalability and cannot withstand DDoS and MITM attacks [81]. To provide efficient privacy preservation and increase network security, a security protocol based on blockchain and IPFS is developed by Meisami [37]. This protocol is shown to minimize cost consumptions in the eHealth system and can mitigate threats such as DoS, modification attacks, and 51 %. However, it is defenseless against spoofing and replay attacks, and cannot offer perfect integrity protection. These security threats crop up when adversaries capture a legitimate patient's data, allowing them to manipulate the permissions of other entities.

Based on content identifier hashes, Batchu et al. [23] proposed a smart contract to store and retrieve medical images using IPFS protocol. However, this technique faces serious problems associated with the implementation of smart contracts, such as the need for solidity expertise. There is therefore need for better and more reliable platforms than Ethereum [23]. To improve authentication, security, and efficiency in IoHT systems, Muwafaq et al. [39] proposed an approach based on Bloom filters, Firebase framework, and Blockchain. In this method, the Bloom filter accelerates authentication, while the Firebase framework enables real-time synchronization. On the other hand, blockchain ensures data security and integrity. Despite its benefits, the Bloom filter is costly, owing to its high resource requirements. In addition, it has high false positives [39].

### Fog computing

To verify, identify, and authenticate IoT devices in fog computing environment, Corthis et al. [29] present a security model that integrates Machine Learning (ML), ECC encryption algorithm, and Proxy *Re*-encryption (PR). In addition, this technique is strengthened by the Enhanced Salp Swarm Algorithm (ESSA). This model effectively identifies and predicts vulnerabilities in the IoHT environment. In addition, it achieves significant improvements in terms of energy consumption, throughput, and response times compared to traditional cryptographic methods. However, the model has not been tested against cyber-attacks and lacks formal security analysis tools [29]. Alzaidi et al. [54] presented a blockchain-based QR code authentication schema in fog-VANETs. It offers privacy and integrity protection without the involvement of a trusted authority. In addition, it blocks attackers, allows anonymous revocation, simplifies conflict resolution, and balances performance with complexity [54].

To build a group authentication for the IoMT framework utilizing fog computing, Norah et al. [38] have employed ECC, Shamir’s secret sharing (SSS) algorithm and blockchain. The system supports scalability and efficiency at low costs, and hence is suitable for resource-limited devices. However, high communication costs are incurred when verifying many devices. In addition, it cannot protect against 51 % attacks. Table 1 compares and contrasts these related works.

**Table 1:** Comparative evaluation of supported functionalities.

Functionality	[41]	[35]	[23]	[28]	[40]	[37]	[38]	[3]	[31]	[17]	[15]	[16]	Ours
Mutual authentication	√	√	√	×	√	√	×	√	×	×	×	×	√
Session key agreement	√	×	√	√	×	√	√	×	×	√	×	×	√
Key security	√	√	×	√	×	√	√	√	√	√	√	√	√
anonymity and confidentiality	√	√	√	×	√	√	×	√	√	√	√	√	√
Formal verification	√	√	√	√	√	√	√	√	√	×	√	√	√
**Security threats**													
DDoS	×	×	√	√	×	√	√	√	–	–	√	–	√
MITM	×	√	√	√	√	×	√	√	–	–	√	–	√
Eavesdropping	√	√	×	√	√	–	√	–	–	–	–	–	√
Replay	√	–	√	–	√	–	√	√	–	–	–	–	√
Spoofing	√	√	×	√	√	–	×	–	–	√	–	–	√
Physical threats	√	√	√	–	–	√	×	–	–	–	–	–	√
Hijacking	×	×	√	–	√	–	√	–	–	–	–	–	√
Sybil	–	–	√	–	–	–	√	–	–	–	–	–	√
51 % attack	–	–	–	–	–	√	×	√	–	–	√	–	√
Quantum threats	–	–	–	–	–	–	–	–	√	√	√	√	√
**Mechanisms**													
Blockchain	×	√	√	×	×	√	√	√	√	×	×	×	√
IPFS	×	√	√	×	×	√	√	×	×	×	×	×	√
Encryption	√	√	√	√	√	√	√	√	√	√	√	√	√
KEM	×	×	×	×	×	×	×	×	√	√	√	√	√
√ supported × not supported - not considered													

Based on Table 1 above, the current works have numerous setbacks, such as security vulnerabilities, high communication and computational costs, as well as lack of support for integrity and scalability. These vulnerabilities can lead to serious problems such as key misuse and data theft, which compromise the health systems and patient health. In addition, most of the current systems may be prone to single point of failure challenge.

This research integrates pre-quantum and post-quantum cryptography techniques such as ECC, CTR, and Kyber-512 to build an authentication and key management scheme for healthcare stakeholders. Through the use of fog computing, blockchain and IPFS technologies, our work complies with privacy and security standards while maintaining integrity and scalability.

## Basics of deployed techniques

This section describes the basic security techniques that are incorporated to realize the functionalities of the proposed scheme.

### Elliptic curve cryptography (ECC)

ECC is a powerful asymmetric key-based technique that secures data through encryption. It generates key pairs (public and private) using the mathematics of elliptic curves and large prime numbers [42]. ECC operations are based on the curve plane over a finite field. The curve consists of points satisfying the following equation:

(1) $$y^{2}=x^{3}+ax+b$$

where {*a, b*} ∈ *F*_p_={0 ,1 ,2, …, *p* −1} and P is the number of the points on the curve. To ensure that the discriminant of the elliptic curve for *a* and *b* is non-zero, Eq. (1) must satisfy the following condition:

(2) $$4a^{3}+27b^{2}\neq 0$$

Eq. (2) is indicative of a non-singular curve [43].

Notably, the public key is chosen as a point on the curve. On the other hand, private key is a random integer, while *G* is a base point on the curve [29]. In the proposed scheme, the ECC algorithm is utilized to encrypt exchanged messages during authentication.

### Counter mode (CTR)

CTR encryption is an efficient and flexible symmetric key encryption technique that converts a block cipher into a stream cipher. To ensure that even the same block, when encrypted again will be different, it generates unique counter and nonce values that are incremented for each block [3]. To get the cipher counter which will be XORed with the plain text block, for each block of data (such as 128 bits), the counter value is incremented and encrypted with the secret key [44].

On the receiver side, the same nonce, key, and counter are used to generate the same counter values. To retrieve the original plaintext, the cipher-text is decrypted by XORing it with the corresponding encrypted counter [44]. In our work, CTR was chosen due to its speed, efficiency, and flexibility. As such, it helps provide security at low resource requirements.

### Key encapsulation mechanism (KEM)

KEM is a secure mechanism used to exchange a symmetric key between two parties using asymmetric algorithms. Its security is based on the Learning-With-Errors (LWE) problem over module lattices. It is considered as one of the post-quantum encryption methods capable of resisting quantum attacks [45]. In KEM, the sender encapsulates the symmetric key within a ciphertext using the recipient’s public key. Upon receiving the cipher text, the recipient decapsulates and retrieves the symmetric key using their private key. This ensures secure and authenticated key exchange without directly sharing the symmetric key during transmission [17].

CRYSTALS Kyber, one of the post-quantum encryption algorithms, was designed based on lattice structures. It is meant to strengthen data security against potential quantum threats [45].

#### Learning-with-errors (LWE)

LWE is a cryptography method based on a mathematical problem that makes it difficult to recover secret information from noisy data samples. Due to its computational complexity, it helps build secure cryptographic algorithms. It renders the recovery of the original information from the processed data extremely difficult [46].

The LWE problem is defined as following:

Given a secret vector *s*:
Generate a uniform vector *a* and noise *e*.
The output *t* = (a*s + *e*) mod *q*.
s,e→χ
sk=s,pk=t=As+e
$r,e_{1},\;e_{2}\;\leftarrow \chi$
$u\leftarrow A^{T}r+e_{1}$
$v\leftarrow \;t^{T}r+\;e_{2}+\mathrm{Enc}\left(m\right)$
c=(u,v)
$m=\mathrm{Dec}\left(v-s^{T}u\right)$
Ex: Let *A*$\in Z_{q}^{m\times n},b=As+e\;and\;s\in Z_{q}^{n},$
m=2n.logq
*A. S* + *e* = *b, q* = 17
$\left[\begin{array}{c} 2\;\;\;13\;\;\;7\;\;3\\ 4\;\;\;\;7\;\;\;9\;\;1\\ 6\;\;14\;\;5\;\;11 \end{array}\right]\times \left[\begin{array}{c} 8\\ 3\\ 12\\ 5 \end{array}\right]+\left[\begin{array}{c} 1\\ -1\\ 2 \end{array}\right]=\left[\begin{array}{c} 2\\ 12\\ 3 \end{array}\right]$

### Crypto hash algorithm 256-bit (SHA-256)

This is a cryptographic hash function developed by the National Security Agency (NSA) that produces a fixed-size 256-bit (32-byte) hash value from input data of any size [47]. It is widely adopted in data encryption protocols such as Secure Sockets Layer, (SSL), Secure Shell (SSH), and blockchain technology. It is majorly used for integrity verification, and digital signatures. Any subtle changes in the input result in drastically different hash outputs. As such, it is an irreversible operation, and hence resistance to collision [48].

SHA-256 pads messages with '1′ then '0′ bits to reach 448 mod 512, appending the original message length in the final 64 bits. The padded message is divided into 512-bit blocks. Each block is expanded into 64 32-bit words, using bitwise operations and modular additions to generate the final 48 words. Using logical functions and constants derived from cube roots of primes, the hash value is updated through 64 rounds of processing. This process produces a final 256-bit hash [49], which can authenticate passwords securely [3]. Theoretically, SHA-256 is better than SHA, which has fewer bits. Therefore, SHA-256 can easily resist brute-force attacks. In overall, it performs well on most devices, balancing between the lower security and speed of SHA-1 and the higher security and computational requirements of SHA-512 [50].

### Blockchain (BC)

**Fig. 1:**
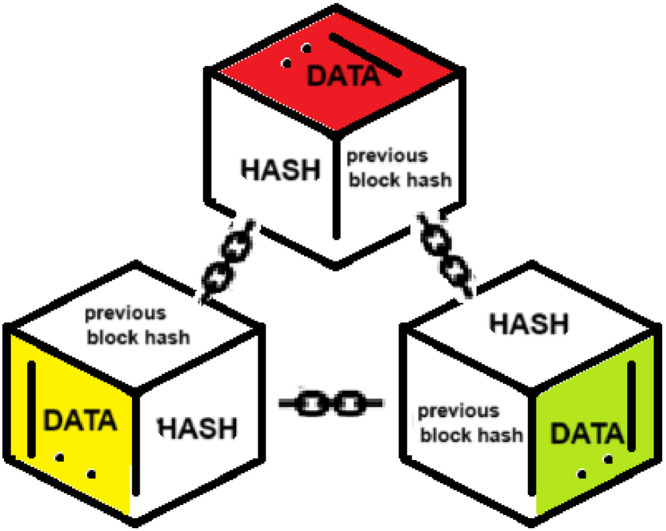
BC blocks connectivity.

BC is a new technology for storing data in connected blocks in form of a decentralized distributed ledger. In BC, each block contains a cryptographic hash of the previous block [51]. This interconnection ensures that any alteration in one block affects all linked blocks, making them resistant to manipulation by individual nodes [52]. Various data in BC are limited and immutable. They include varied data types such as currency and medical information. The BC’s distributed and decentralized architecture protects the system from single point of failure problems [24]. This renders it an ideal choice for storing data hashes in the proposed scheme. Fig. 1 gives an illustration of BC connectivity.

### Interplanetary file system (IPFS)

IPFS is a decentralized distributed network specifically designed for peer-to-peer file storage. Instead of a single server, files are divided into smaller chunks, hashed, and stored across multiple nodes. This decentralization boosts robustness, protecting against censorship and data loss [2]. This innovative system is based on content-based processing principles. To search for any file, unique cryptographic hashes are generated directly from the file content itself instead of relying on traditional location-based processing. These cryptographic hashes can be considered as an alternative or surrogate representation of the file content. This ensures that the retrieved data matches what was requested. In addition, it helps ensure integrity, as any change in the content results in a different hash [21].

**Fig. 2:**
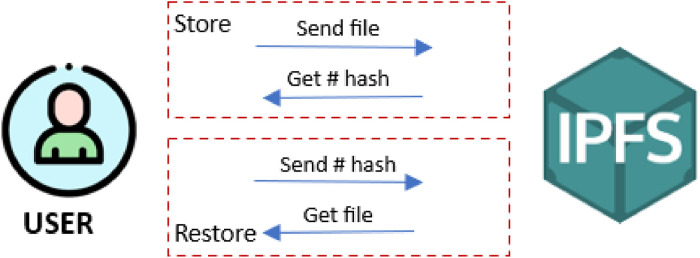
Mechanism of storing data in IPFS.

As shown in Fig. 2, the file storage mechanism works in a way that allows data to be retrieved and stored efficiently across the network. All that is needed is to query IPFS network for the file's hash. IPFS locates the nearest nodes that have the file and fetches the data from them. The data is then reconstructed on the user devices.

### Fog computing FC

FC is a decentralized infrastructure that enhances cloud computing by bringing data processing, storage, and application services closer to the network edge. Since operations are performed nearer the data sources such as sensors, smart devices, and local servers, FC improves efficiency and minimizes latencies. This is beneficial for many services, especially with real-time applications [53]. Moreover, FC minimizes bandwidth usage and lowers the burden on the network. In addition, it supports scalability by facilitating easy addition and management of devices. Moreover, processing data locally reduces exposure to potential breaches and improves data privacy. FC is particularly suitable for IoT systems such as smart cities, eHealth systems, and industrial automation where real-time responses and low latency are critical [54]. The FC consists of three layers:

• **Things layer:** This layer consists of connected devices that act as data inputs. Such devices include as sensors, wearable devices, Arduino, and actuators [53].
• **Fog layer**: This is the middle layer whose task is transferring data between data collectors (things layer) and the cloud. It includes network devices, Internet gateways, local servers, and routers [54].
• **Cloud layer:** This is the third layer which receives and processes data using servers with high processing and storage capabilities. These servers have remarkable abilities in analyzing data to facilitate decision making [55].

FC complements, rather than replace cloud computing. Certain data may still be sent to the cloud for long-term storage, complex analytics, or non-urgent processing [54,55].

### Searchable symmetric encryption (SSE)

SSE is a powerful encryption method for storing and retrieving encrypted data on a remote server or fog node while preserving data privacy. This technique utilizes symmetric encryption and is ideal for handling large databases. It is simple, secure, searchable, and cost-effective [56]. SSE achieves significant improvements in information retrieval performance by improving locality. This is accomplished by changing the storage mechanism of the encrypted inverted index [57].

SSE is resistant to several attacks, such as keyword guessing and frequency analysis. SSE starts with the key generation process, which is implemented by the data owner. Thereafter, it creates a secure index to the cloud server. To search for specific data, a token is created by the user and sent to cloud server for searching so as to find the required data and acquire the identifiers related to it [57].

## Security goals and threats

Data protection in IoHT systems is a key focus in healthcare studies due to concerns over health data privacy, device reliability, and overall system security [58].

### The goals of the proposed work

The key goals that the proposed low-complexity secure IoHT must meet are based in some well-known security requirements:

• **Authentication:** This process allows IoHT system entities to identify their peers with whom they interact and grant permission to access system resources [59].
• **Authorization**: Ensures that only authenticated parties have access to system resources and are assigned their tasks based on their roles [60].
• **Integrity**: This is the process of ensuring that data exchanged between system components and stored is valid and has not been tampered with [61].
• **Confidentiality**: Ensuring that the medical data is protected, immutable, and accessible only by authorized users [2].
• **Availability**: Ensures the effectiveness and the continuity provision of healthcare services. It guarantees that medical devices and data are accessible anytime and anywhere [2,62].
• **Non-repudiation:** Ensures the credibility of the message source (device or user) and prevents this source from denying its transmission [63].
• **Privacy:** Deals with the process of ensuring that personal and sensitive information is not exposed to leakages or unauthorized access. It also guarantees that individuals are granted control over their information [64,65].
• **Forward secrecy**: It ensures that if the session key of a certain session is compromised, it would not jeopardize subsequent sessions. To achieve this, each session is protected with a unique and invulnerable key [66].
• **Backward secrecy**: If an attacker can breach a long-term key for all sessions, it must be infeasible for the attacker to use it to decrypt previous, current, or future messages [14].
• **Unlinkability:** It deals with keeping transactions and actions from being tracked or associated with undesired entities [3].
• **Secure key management:** It involves the generation, storage, distribution, and safeguarding of encryption keys [25].

### Security threats

With the continuous development of the IoHT infrastructure, it may be vulnerable to cyber-attacks and threats. This can threaten the safety of patients and availability of the health systems through techniques such as brute-force and dictionary attacks. These attacks use powerful devices and software to test numerous password combinations and encryption keys to gain unauthorized access. Online dictionary attacks involve attempting to log in directly to the system within allowed attempts, while offline dictionary attacks use a hashed password database, enabling unlimited attempts on the hashes [3]. On the other hand, MITM attacks remotely intercept and manipulate data during the communication process [23].

Quantum attacks exploit quantum computers' powerful computational capabilities over classical computers to violate security [45]. On the other hand, eavesdroppers intercept unencrypted data during transition, potentially collecting or altering it [66]. Regarding spoofing attacks, adversaries impersonate authorized devices or accounts (such as IP, and DNS) to steal, sabotage data or resources [67]. On the other hand, DDoS attacks overwhelm systems with malicious requests, rendering them inaccessible [68,69]. On their part, Sybil attacks involve a single-user node appearing with multiple identities. This user deploys malware to steal data by impersonating a legitimate user [70]. On the other hand, physical attacks target the network infrastructure, disrupting functions, destroying devices, and causing them to go out of service. In addition, physical attacks can lead to data lose or leakages [71].

In node capture attacks can enable adversaries to gain access to a crucial system or group of nodes to steal data stored on IoHT network devices [72]. On the other hand, timing attacks try to find weaknesses in the devices that can facilitate hacking. This is achieved by tracking and analyzing the device response time [73]. Concerning impersonation attacks, it involves an adversary masquerading as a trusted entity to access data and information belonging to patients and systems [75]. On the other hand, replay attacks are executed by capturing and reusing valid authentication messages to gain unauthorized access [61]. However, hijacking attacks involve adversaries trying to obtain or guess the session key of an authorized user and use it to gain unauthorized access to the user's session [76]. On the other hand, 51 % attack occurs when malicious users control over 50 % of a network. This allows them to alter records, halt confirmations, and block payments and transactions [77]. Finally, phishing attacks involve stealing personal user data such as logins and credit cards. This is attained through deceptive communications as well as impersonation of trusted entities [78].

## Proposed scheme details

**Fig. 3:**
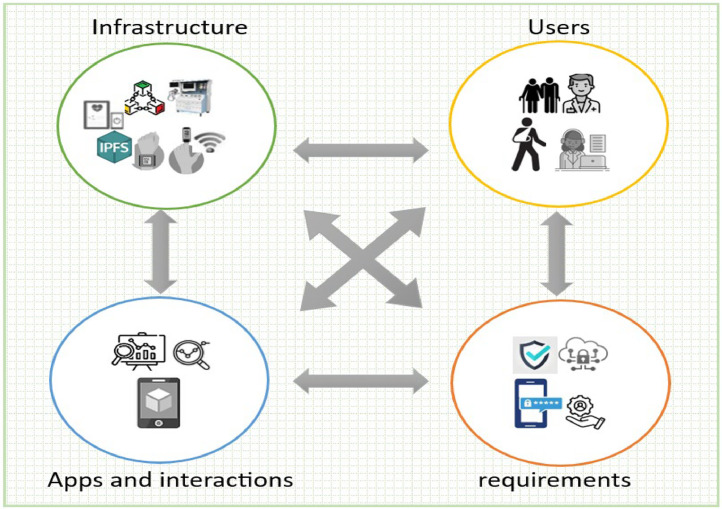
IoHT components.

The proposed scheme is realized by the interaction of several key components that cooperate to ensure the safety of patient data, while allowing secure and efficient access from anywhere and at any time by authorized entities. It also provides access during emergencies and critical patient cases, while maintaining data privacy and security. The interaction among the various network elements in the proposed scheme is illustrated in Fig. 3. The function of each of these network elements is described below.

*Infrastructure:* consists of the physical and cloud components such as sensors, wearable devices, fog nodes, cloud storage, EHR, BC, IPFS, as well as access and management devices.

*Users:* represents a group of people and institutions that work, interact, and benefit from the system directly or indirectly. This includes patients, their appointees, administrators, system operators, data entry clerks, healthcare institutions, doctors, health service providers, ambulance operators, and emergency services. Table 2 presents different types of users, including their roles.

**Table 2:** Role-based access control for IoHT’s entities.

Users	Definition	Role	Authority
Cloud Health Service Provider CHSP	Supervisors and Service Providers	Organizing and controlling access permissions, registering administrators and generating keys	Read/ Write / Edit
ADM	Health system administrator and supervisors	Register, manage, direct and assign roles to all system components	Read / Write/ Edit
Patients	The group of people targeted by the system and to be served	Create a medical record, register their devices	Read only
Medical staff	The medical professional persons like: doctors, nurses	Access patient records, enter medical information, and provide health care.	Read / Write

*Applications and interactions*: This refers to a collection of programs, applications, and processes designed to manage the system, data, monitoring, and services provision. The proposed scheme comprises of the following major phases: (1) setup and registration, (2) RBAC authentication (3) secure data exchanging (4) data updating.

*Functionality and security requirements:* This is a set of security measures aimed at securing data and managing authorizations. The essential functionalities in our scheme include key agreement and distribution which based on key generation KGen(α), registration, data encryption and decryption $Enc_{\beta }/Dec_{\beta }\left(data\right)$, where β is the Key of encryption, as well as hybrid algorithm for admin and data securing using Kyber-512 algorithm, ECC, SHA-256 and symmetric algorithm CTR.

**Fig. 4:**
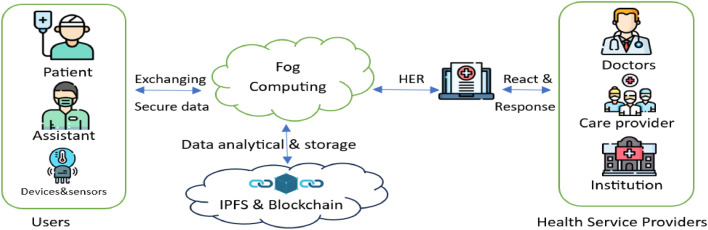
Proposed scheme components.

Fig. 4 illustrates the components of the proposed scheme, which is an innovative secure IoHT system designed to effectively manage patient data and provide immediate responses, especially in emergencies or critical conditions. The scheme robustly encrypts and secures data, which is then stored in EHR on fog nodes, IPFS and Blockchain. Data entry, modification, and access are restricted for patients, assistants, and data entry employees. This ensures that medical staff, doctors, and healthcare institutions can promptly retrieve necessary information. The system entities’ interactions within the proposed scheme include registration, authentication, key exchange and negotiation, as well as role-based authorization. Table 3 details the symbols used throughout this paper.

**Table 3:** List of symbols.

Symbol	Description
*PuH*	Public key
*PrH*	Private key
*CHSP*	Cloud Healthcare Service Provider
*ADM*	Administrator
*SK_i_*	Shared symmetric key
*Pu_ADMi_,Pr_ADMi_*	*ADM’s* keypairs
*ADM_iinfo_*	Administrator registration request
*AN_i_*	Username
*PW_i_*	Password
*Hash function*	*h*()
*DH_ADMi_*	Hash address in IPFS
*T1*	Timestamp
*R_1_*	Random nonce
⊕	XOR operation
*VC*	Verification Code
*SK_it_*	One-session secret key
*Enc / Dec ()*	Encryption and decryption operations
*SK_pi_*	Secret key for patient
KGen(α)	Key generation algorithm

### Setup phase

In this phase, Cloud Healthcare Service Provider (*CHSP*) is responsible for creating main keys utilized in the subsequent phases. The *CHSP* initializes the operators of the ECC as follows

**Step 1:** Select *P* and *G* as a large prime numbers. Here, *P* is the number of the points on the curve while *G* is a base point on the curve.

**Step 2:** Choose {*a, b*} ∈ *F*_p_={0 ,1 ,2, …, *p*-1}, where *F*_p_ is the finite field over the elliptic curve. Next, *CHSP* randomly selects a private key (*PrH*) within the range of curve points.
**Step 3:** The *CHSP* evaluates the PuH←KGen(PuH) to calculate the public key (*PuH*) based on *PrH* as PuH= PrH * G. These key pairs (*PuH, PrH*) are used for ECC and KEM during registration and for exchanging the symmetric key (*SK*) between CHSP and *ADM_i_*.

The same steps are followed to generate key pairs for *ADMs*. Basically, CHSP initiates key pairs (*Pu_ADMi_, Pr_ADMi_*) for *ADMs* and key pairs for any other users, which are then deployed during their registration phase.

*ADM registration phase*

**Fig. 5:**
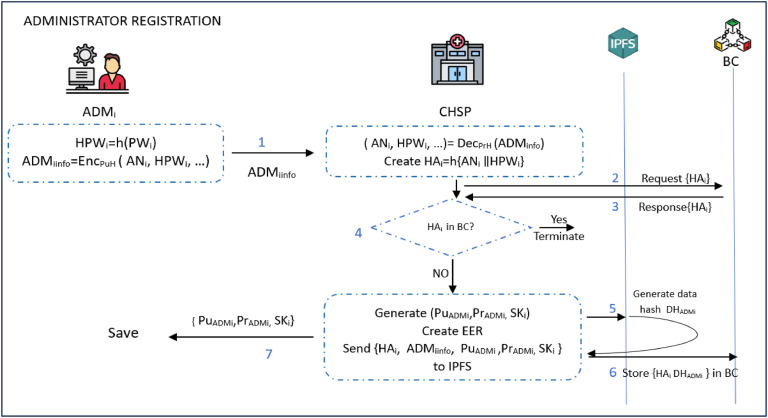
Block diagram of the ADM registration mechanism.

The goal of this phase is to register the ADM to the *CHSP*. This is accomplished by following the steps below:

**Step 1:** The *ADM*_i_ creates a registration request, which includes important information such as the user’s name *AN*_i_, hashed password *HPW_i_*= *h* (*PW*_i_), and phone number *PH*_i_. This request is then encrypted using the *CHSP*’s public key *PuH* as *ADM_iinfo_= Enc_PuH_* (*AN_i_* ∥*HPW_i_* ∥*PH_i_*)
**Step 2:** On getting request *ADM_iinfo_*, the *CHSP* decrypts it using its private key *PrH* to get the full information as (*AN_i_, HPW_i_*)*= Dec_PrH_* (*ADM_iinfo_*). It then creates *HA_i_=h{ AN_i_,* ∥ *HPW_i_ }* and sends it to *BC*. Provided that *ADM*_i_ has not been registered previously, *CHSP g*enerates asymmetric and symmetric keys (public key, *Pu_ADMi_*), private key (*Pr_ADMi_*), shared key (*SK_i_*)).
**Step 3:** The *CHSP g*enerates an Electronic Employee Record (EER) containing all the previously derived *ADM*_i_ information and associated keys. It then sends {*HA_i_, ADM_iinfo_, Pu_ADMi_ ,Pr_ADMi_* , *SK_i_* }to IPFS and retrieves its hash address (*DH_ADMi_*). Next, it stores { *HA_i_* , *DH_ADMi_* } in BC. At the end, it sends (*Pu_ADMi_* , *Pr_ADMi_, SK_i_* ) to the *ADM*_i_ via a secure channel, where they are store in his/her device. Fig. 5 gives a pictorial representation of these processes.

### ADM login and authentication phase

This phase is activated when *ADM*_i_ intends to obtain access to the CHSP services, and servers. To achieve this, the following steps are performed.

**Fig. 6:**
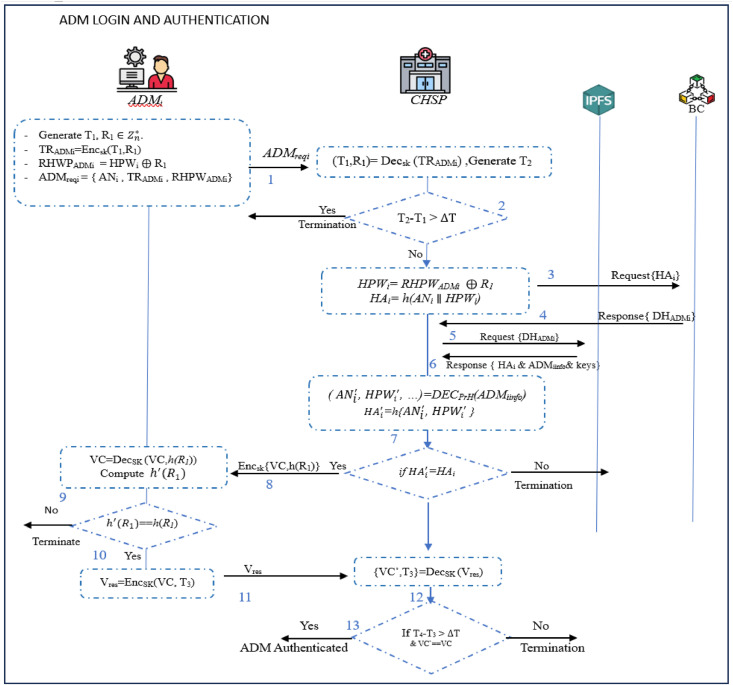
Login and authentication phase for ADM.

**Step 1:** The *ADM_i_* computes the authentication request by generating a timestamp and random nonce *T_1_, R_1_* $\in Z_{n}^{*}$, respectively. Next, he/she derives *TR_ADMi_=Enc_sk_* (*T_1_,R_1_*), *RHPW_ADMi_ = HPW_i_* ⊕ *R_1_* and *ADM_reqi_ =* {*AN_i_ , TR_ADMi_ , RHPW_ADMi_*}. Finally, it sends *ADM_reqi_* to *CHSP*, that is, *ADM_i_* →*CHSP : ADM_reqi_*
**Step 2:** On receiving *ADM_reqi_*, the *CHSP* computes (*T_1_,R_1_*)*= Dec_ski_* (*TR_ADMi_*). Next, it generates timestamp *T_2_* and checks if |*T_2_-T_1_*| *>* ΔT*,* where ΔT is the permissible transmission delay. Provided that this condition does not hold, a *time out* message is generated and the session is terminated. Otherwise, the *CHSP* calculates *HPW_i_= RHPW_ADMi_* ⊕ *R_1_* and *HA_i_= h*(*AN_i_* ∥ *HPW_i_*). At the end, it sends verification request to *BC* with *HA_i_.* If this request is successfully verified, the *BC* responds with *DH_ADMi_*.
**Step 3:** Using *DH_ADMi_*, information (*HA_i_, ADM_iinfo_, Pu_ADMi_, Pr_ADMi_* , *Sk_i_*) is rereived at IPFS node. It then retrieves the *ADM_iinfo_* through decryption using *PrH*. This is followed by the creation of $HA_{i}^{\prime }$ with hash-crypto *h*(.) as $HA_{i}^{\prime }$*=h* ($AN_{i}^{\prime }∥HPW_{i}^{\prime }$). It then confirms whether $HA_{i}^{\prime }$ *= HA_i_*, terminating the session is this condition does not hold. Otherwise, it generates Verification Code (*VC*) and *h*(*R_1_*), both encrypted with *SK_i_* . Finally, both are sent to *ADM_i_* as *CHSP* → *ADM_i_ :* {*VC , h* (*R_1_*)}*_SK_.*
**Step 4:** After getting ghe above message, the *ADM_i_* performs decryption as *Dec_Sk_* (*VC, h*(*R_1_*)) and to regain *VC* and *h*(*R_1_*). He/she then computes $h^{\prime }\left(R_{1}\right)$ and checks if $h^{\prime }\left(R_{1}\right)$=*h* (*R_1_*). Provided that this condition does not hold, the *ADM_i_* suspends the session. Otherwise, he/she generates timestamp *T*_3_ and creates response message *V_res_=Enc_SK_* (*VC, T_3_*) that is sent to *CHSP* as *ADM_i_* → *CHSP : V_res_*
**Step 5:** Upon obtaining *V_res_*, the *CHSP* retrives and verifies *VC’* and *T_3_*. If this veirification is successful, *ADM_i_* is regarded as being authenticated. Otherwise, the session terminated. Fig. 6 summarizes these login and authentication procedures.

### Secret key sharing phase

The secret key exchange is an influential and critical step for securing the data exchange between the *ADM* and *CHSP*. Once an authentication session is established, the connected parties (that is, *CHSP* and *ADM*) typically exchange a session secret key (*SK_it_*). To ensure robust security, this randomly chosen symmetric key is valid for only one session. Utilizing a unique random session key enhances the system's resistance to various cyber-attacks, including dictionary, brute-force, guessing, and replay attacks. In addition, it maintains forward and backward secrecy.

Therefore, the key is exchanged securely using the KEM technique based on the Kyber-512 encryption algorithm. The following steps are executed to achieve this secret key sharing.

**Fig. 7:**
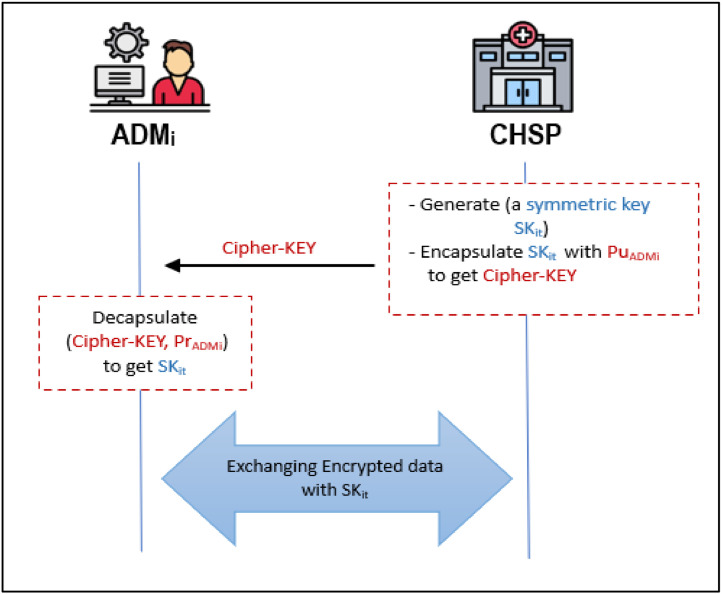
Secret key generation and exchanging phase.

**Step 1:** The *CHSP* generates a random large prime number *S*, and timestamp *T_i_.* It then calculates the secret shared key as *SK_it_= h*(*S*∥*T_i,_*∥*SK_i_* )*.* This is followed by the encapsulation of *SK_it_* with Kyber algorithm and *Pu_ADMi_* to get a *Cipher-KEY*. Finally, it sends *Cipher-KEY* to the *ADM_i_*.
**Step 2:** After receiving *Cipher-KEY*, the *ADM_i_* decapsulates the *Cipher-KEY* with his/her private key *Pr_ADMi_* to retrieve *SK_it_*. A summary of these key sharing procedures are illustrated in Fig. 7.

Patients are among the most important entities in a healthcare association. In the following sub-sections, we describe the patient registration, as well as authentication procedures.

### Patient registration phase

All patients must be registered to enable the system manage the EHR and monitor the patient's health and vital signs. Patient registration processes involves registering the patient as well registering his/her personal devices. The steps that need to be performed during the patient registration phase are described below.

**Fig. 8:**
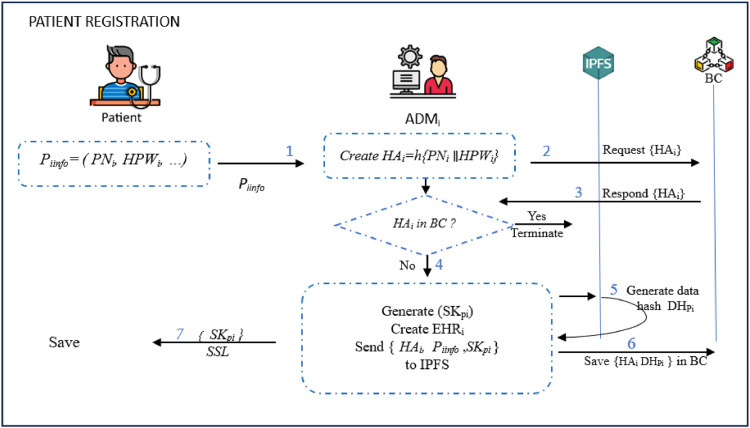
Patient registration phase.

**Step 1:** The patient *P_i_* sends his/her request to the *ADM_i_* including patient name *PN_i_, HPW_i_=h*(*PW_i_*)), phone no *PH_i_*, and address *ADD_i_* via a secure channel as *P_iinfo_→ ADM_i_ : P_iinfo_ =* {*PN_i_, HPW_i_, PH_i_, ADD_i_*}.
**Step 2:** On receiving *P_iinfo_*, the *ADM_i_* creates *HA_i_=h* (*PN_i_* ∥*HPW_i_*) and sends a request to *BC*. Basically, checks are performed to establish whether this patient had already been previously registered. On condition that this is the case, the session is terminated. Otherwise, *ADM_i_* generates shared keys *SK_pi_* for the *P_i._*. This is followed by the generation of Electronic Health Record (EHR) containing *Pi* information and associated keys.
**Step 3:** The *ADM_i_* sends {*HA_i_, P_iinfo_ ,SK_pi_* } to IPFS and retrieves its hash identifier (*DH_Pi_*). It then saves {*HA_i_, DH_Pi_*} in *BC* before sending the shared keys to the *P_i_* via a secure channel (SSL) as *ADM*_i_ → *P*_i_: {*SK_pi_*}
**Step 4:** After receiving *SK_pi_, P_i_* stores them in his/her device and confirms the registration. Fig. 8 summarizes these patient registration procedures.

**Fig. 9:**
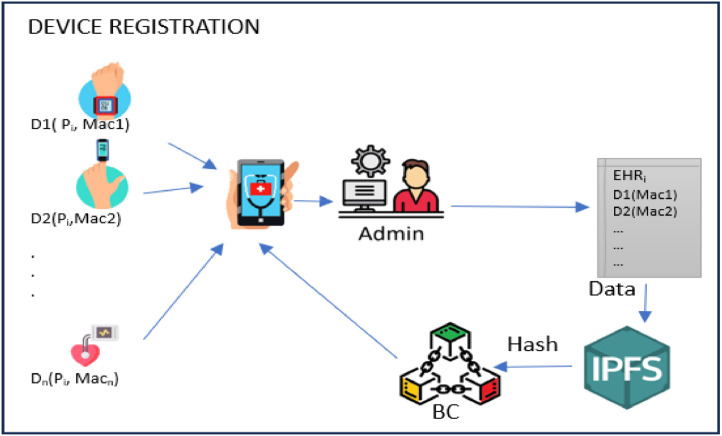
Device registration phase.

After successful patient registration, the medical devices registration phase is activated. The *ADM_i_* is responsible for registring the *P*_i_ related devices that used to collect patient vital data. This registration is accomplished by assigning an identifier to each device. This identifier comprises of two values: the patient's name and the device's Media Access Control address (MAC) and creates a devices list in *EHR_i_*. This ensures that only the device owner can register them, protecting against intrusive or malicious devices that might transmit incorrect data. In this way, integrity and confidentiality are attained. After registering, the data collected from the devices is encrypted with a symmetric key *SK*_pi_ stored in the *EHR*_i_ of the patient. Thereafter, it is forwarded to the IPFS where its hashes are uploaded to the Blockchain. The medical devices registration procedures are summarized in Fig. 9.

### Devices re-authentication phase

The procedures executed in this re-autehentication prevents unauthorized access and ensures that only legitimate devices can communicate within the network. IoT devices often move among networks. This re-authentication is occassioned by Internet disconnection, switching off of the device due to low battery, or detection of abnormal behavior. It ensure that secure communications are maintained [74,75].

The *Node_Pi_* (patient mobile app) handles frequent re-authentication, triggered by sensor signal loss (due to connection drops or power failures). It acts as a fog node to reduce computational load on other nodes. Basically, *Node_Pi_* continuously reads data from the sensors to monitor the patient’s condition, analyzes the signals, and responds to any abnormal or sensitive readings. This is performed with varying reading intervals based on the significance of the sensed signal. This re-authentication is accomplished by performing the following procedures.

**Fig. 10:**
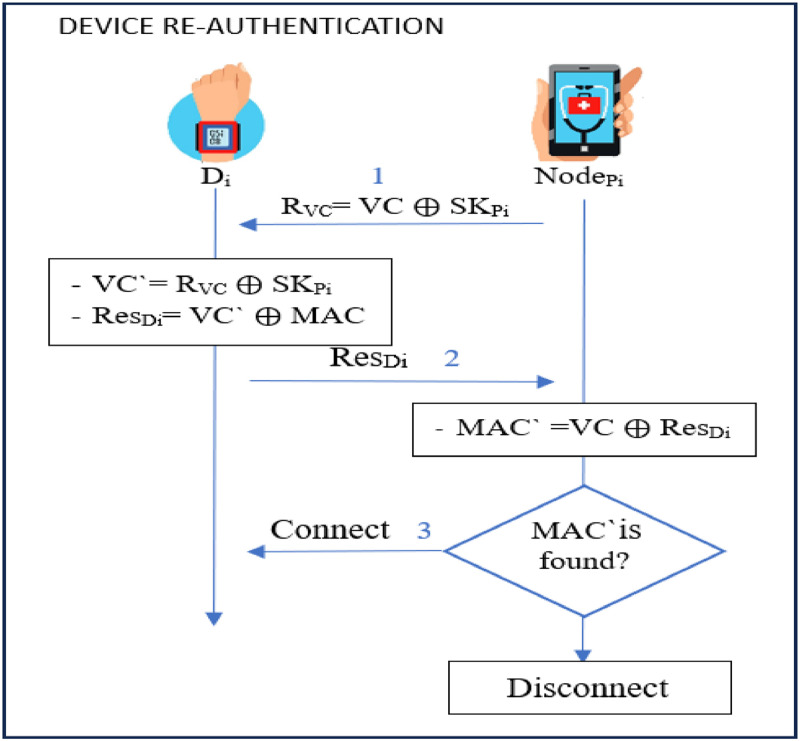
Fog node (Node_pi_) and device re-authentication phase.

**Step 1:** Upon detection of signal loss from sensor device *D_i_, Node_Pi_* generates *R_VC_ = VC* ⊕ *SK_Pi_*. It then sendi *R_VC_* to *D_i_* as *Node_Pi_ → D_i_ : R_VC_*
**Step 2:** On getting *R_VC_, D_i_* derives *VC`= R_VC_ ⨁ SK_Pi_* and *Res_Di_= VC` ⨁ MAC*. At the end, it sends *Res_Di_* to *Node_Pi_* as *D_i_ → Node_Pi_ : Res_Di_*.
**Step 3:** After receiving *Res_Di_, Node_Pi_* computes *MAC` =VC* ⊕ *Res_Di_* and utilizes a secure search algorithm SSE [57] to look for the MAC address in the trusted list. This lowers retrieval costs and improves search efficiency. If this MAC adddress is found, the connection is maintained. Otherwise, disconnection is performed. Fig. 10 gives an illustration of these procedures.

### Patient login and authentication phase

Authentication ensures secure access to health systems and vital data. In addition, it prevents attackers and unauthorized entities from invading the network and its devices. In so doing, it protects user privacy, and establishes accountability. To authenticate a patient, the following procedures are carried out.

**Fig. 11:**
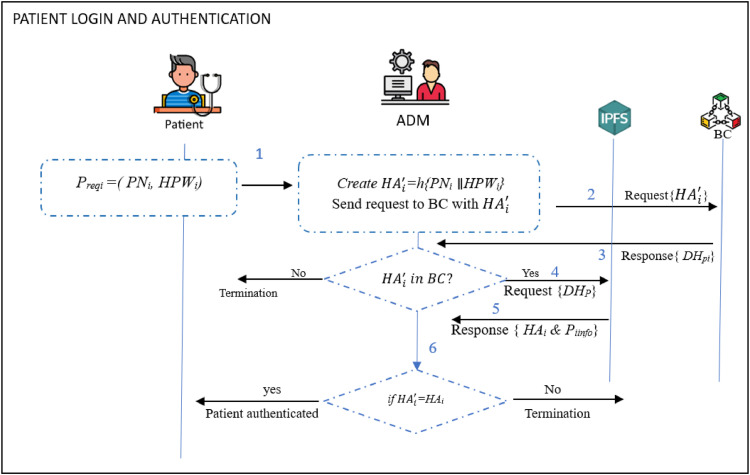
Patient login and authentication process.

**Step 1:** The patient *P_i_* sends his/her login request to the *ADM_i_*. This request includes *PN_i_* and *PW_i_* (contained in parameter *HPW_i_* =*h* (*PW_i_*)) as *P_reqi_ → ADM_i_*: (*PN_i_, HPW_i_*).
**Step 2:** On getting the above values, the *ADM_i_* creates $HA_{i}^{\prime }$*=h* (*PN_i_* ∥ *HPW_i_*). Next, it sends it to *BC* where checks are done to determine whether it exists in the *BC* or not. Once this confirmation is positive, the *ADM_i_* retrieves {$HA_{i}^{\prime }$, *DH_Pi_*} from *BC*, and {*HA_i_, P_iinfo_*} from IPFS using *DH_Pi_*. It then checks if *HA_i_* = $HA_{i}^{\prime }$ such that the login request is denied upon verification failure. Otherwise, the *P_i_* login and authenticated are regarded as being successful. Fig. 11 gives the pictorial representation of these procedures.

In the proposed scheme, the doctor has the access right to read and modify the data in the EHR so as to monitor the patient's condition. Since this directly affects patient's health and the system's security, all dotors need to register and authenticate to the system. Basically, octor *Do*_i_ has three interactions with the system, including registration, authentication, and data editing. The following sub-sections give detailed descriptions of these processes.

### The doctor registration phase

The doctor *Do_i_* registration is implemented by performing the following steps.

**Fig. 12:**
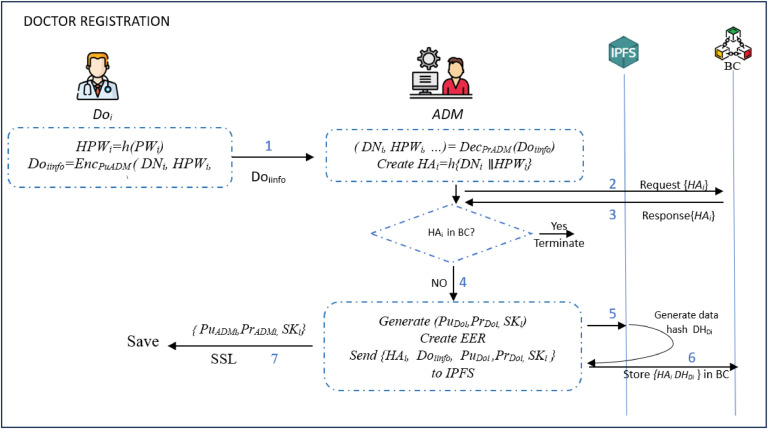
Doctor registration phase.

**Step 1:** The *Do*_i_ creates a registration request that includes registration information such as the doctor’s name *DN*_i_, password (in parameter *HPW_i_*= *h*(*PW*_i_)), and phone number *PH*_i_. These values are then encrypted using the *ADM*’s public key *Pu_ADM_* as *Do_iinfo_= Enc_PuADM_* (*DN_i_, HPW_i_, PH_i_*). At the end, *Do_iinfo_* is sent to *ADM_i_* as *Do_i_ → ADM_i_: D_iinfo_*
**Step 2:** After receiving *Do_iinfo_*, the *ADM*_i_ decrypts the request with its private key *Pr_ADM_* to get the values (*DN_i_, HPW_i_, PH_i_*)*= Dec_PrADM_* (*Do_iinfo_*). It then derives *HA_i_=h* (*DN_i_* ∥ *HPW_i_*) and sends it to BC to confirm whether it had been registered previously. Once this confirmation succeeds, it generates asymmetric and symmetric keys (public key (*Pu_Di_*), private key(*Pr_Di_*), shared key (*SK_i_*)).
**Step 3:** The *ADM*_i_ generates an Electronic Employee Record (EER) containing all the previously mentioned *Do*_i_ information and associated keys. It then composes {*HA_i_, Do_iinfo_, Pu_Di_ ,Pr_Di_* , *SK_i_* } that is sent to IPFS , which then retrieves its hash address *DH_Doi_*. Next, it stores {*HA_i_* , *DH_Doi_*} in *BC* and sends (*Pu_Doi_* , *Pr_Doi_, SK_i_* ) to the *Do*_i_ via a secure channel as *ADM*_i_ → *Do*_i_: (*Pu_Doi_, Pr_Doi_, SK_i_*).
**Step 4:** Upon receiving the above values, *Do*_i_ stores them in his/her smart devices and confirms the registration as illustrated in Fig. 12.

### Doctor login and authentication phase

This phase is initiated when *Do_i_* wants to access to the patient’ records in EHR, system services, and servers. It involves the execution of the following procedures.

**Fig. 13:**
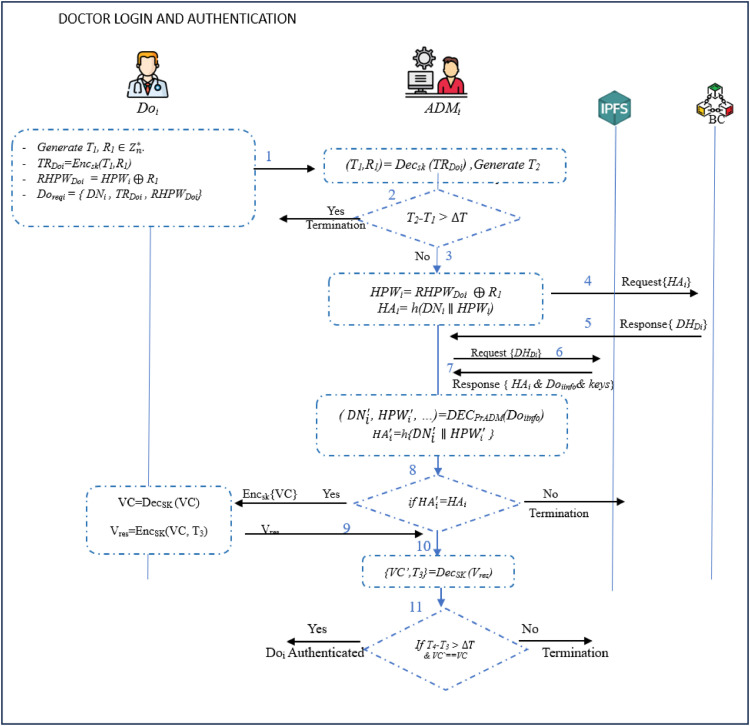
Doctor login and authentication phase.
**Step 6:** On getting *V_res_, ADM_i_* retrieves *VC’* and *T_3_* and compares them with his values. If there is a match, *Do_i_* is successfully authenticated. Otherwise, the session is terminated.

**Step 1:** The *Do_i_* initiates the authentication generating timestamp *T_1_*, random value *R_1_* $\in Z_{n}^{*}$. This is followed by the computation of *TR_Doi_=Enc_sk_* (*T_1_, R_1_*), *RHWP_Doi_ = HWP_i_* ⊕ *R_1_*, and *D_reqi_ =* {*DN_i_ , TR_Doi_ , RHWP_Doi_*}. He/she then sends *D_reqi_* to *ADM_i_* as *Do_i_* →*ADM_i_ : D_reqi_* as shown in Fig. 12.
**Step 2:** After receiving *D_reqi_*, the *ADM_i_* computes (*T_1_, R_1_*)*= Dec_ski_* (*TR_Doi_*) and determines the current timestamp *T_2_*. It then confirms whether |*T_2_-T_1_* |*>* ΔT, generating *time out* and halting the session if this condition is true. Otherwise, it derives *HWP_i_= RHWP_Doi_* ⊕ *R_1_* and *HA_i_= h*(*DN_i_* ∥ *HWP_i_*). At the end, it forwards the forwards a verification request to *BC* with *HA_i_.* If there is a matche, *BC* responses with *DH_Doi_*.
**Step 3:** Using *DH_Doi_*, information (*HA_i_, Do_iinfo_, Pu_Doi_,Pr_Doi_* , *Sk_i_*) is retrieved at the IPFS node. In addition, it retrieves *Do_iinfo_* through decryption by *Pr_ADM_*. This is followed by the creation of $HA_{i}^{\prime }$ using hash-crypto *h*(.) as $HA_{i}^{\prime }$*=h* ($DN_{i}^{\prime }∥HPW_{i}^{\prime }$).
**Step 4:** The *ADM_i_* checks if $HA_{i}^{\prime }$ *= HA_i_*, terminating the session is these two values are dissimilar. Otherwise, it generates Verification Code (*VC*) and sends it to *Do_i_* together with *SK_i_* as *ADM_i_* → *Do_i_:* {*VC , SK_i_*}.
**Step 5:** Upon receiving the above values, *Do_i_* performs decryption as *Dec_Sk_* (*VC*) to retrieve *VC*. It then generates *T*_3_ and creates a response message as *V_res_=Enc_SK_* (*VC, T_3_*). Finally, *V_res_* is sent to *ADM_i_* as *Do_i_* → *ADM_i_: V_res_* as shown in Fig. 13.

## Method validation

### Performance analysis

In this section, we present the simulation environment, and the results of the performance evaluations in terms of latency, throughput, energy consumption, as well as computational and communication costs. The simulation was implemented on machine running a 64-bit Windows OS, equipped with 16 GB of RAM and a Dual-Core Intel Core i7 CPU 13700H operating at 2.40 GHz. The brief description of the deployed performance metrics is given below.

#### Latency

This measures a system's request processing time. Basically, it evaluates responsiveness and speed. It is measured in milliseconds (ms) and is mathematically expressed as follows.

Latency=Responsetime−Submittime

The average latency for the *ADM* registering process was 0.9 ms, while the average latency time for *ADM* login and authentication operations ranged from 1.28 ms to 2 ms.

#### Throughput

It measures the amount of data transmitted between two points within a specific timeframe. It is typically expressed as bits, bytes, or transactions per second (TPS). Mathematically, throughput is calculated as follows.

$$Throughput=\frac{number\;of\;transuctions}{time\;in\;seconds}$$

The throughput of the *ADM* registration phase was in the range [900–1000] TPS. On the other hand, the *ADM* login and authentication throughput increased slightly during the experiment to reach 380 TPS.

#### Computational and communication costs

These metrics are used for measuring temporal complexity, evaluating the proposed schemes' efficiency and cost. Table 4 shows the notations of the various cryptographic operations performed by the proposed scheme’s components.

**Table 4:** Cryptographic runtimes.

Symbols	Operation	Time (Ms)
*T*_h_	One-way hash function	0.7
*T*_ECC_	ECC algorithm	0.8
*T*_CTR_	CTR algorithm	0.14
*T*_Kpr_	Key sharing with KEM	0.7

There are some other operations such as XOR and concatenation but they are neglected due to their extremely short execution durations. Table 5 shows the computational time of the various system entities during registration and authentication procedures.

**Table 5:** Computation cost of the overall scheme.

User	Roles	Operations	Total time (Ms)	Time (ms)
ADM	Registration	2*T*_h_+*T*_ECC_	2.2	5.42
	Authentication	3*T*_h_ + 3*T*_CTR_	2.52	
	Key sharing	*T*_Kpr_	0.7	
Doctor	Registration	2*T*_h_ + *T*_ECC_	2.2	4.72
	Authentication	3 *T*_CTR_+ 3*T*_h_	2.52	
Patient	Registration	2*T*_h_	1.4	2.8
	Authentication	2*T*_h_	1.4	
				12.94

To calculate the communication cost of the proposed scheme, we need to determine the length of its messages. Here. We take the length of the hash function to be 256 bits, the random numbers and timestamps are 32 bits, while the length of encryption keys (public, private, and symmetric) are 192 bits, 192 bits and 32 bits respectively. As such, the sizes of the values in registration information (*AN, HPw, Email, phone*) are 64 bits, 256 bits, 128 bits, and 44 bits respectively. In addition, the verification code *VC* is 64 bits. Therefore, the total communication cost for registration is 908 bits.

The login and authentication phase is done more frequently than other phases. Therefore, we need to calculate and compare its communication overheads. In the login phase, *ADM* sends message {*AN_i_, TR_ADMi_, RHPW_i_*} to the *CHSP*. Its communication cost is (64+(32+32)+256) = 384 bits. During authentication, the *CHSP* sends {*VC, h(R)*}, which needs (64+256) =320 bits. Afterwards, the *ADM* replies with (*VC, T*) which requires (64+32) = 96 bits. As such, the overall communication cost is 800 bits.

To measure the system's efficiency, we compared it with other related works in terms of communication and computation costs. Table 6 shows how the proposed system compares to other works.

**Table 6:** Computation and communication cost comparison.

Schemes	Registration	Authentication	Total (ms)	Total (bits)	Energy (mJ)
[28]	2*T*_ECC_+4*T*_h_+*T*_ED_	3*T*_ECC_+10*T*_h_+3*T*_ED_	35.4	1024	0.2336
[40]	12*T*_h_	7T_h_ + 12T_h_	21.7	2248	0.1432
[30]	2*T*_ECC_+4*T*_h_ +*T*_ED_	(3*T*_ECC_+10*T*_h_+3*T*_ED_ ) + (4*T*_ECC_+8*T*_h_)	38.8	1328	0.2561
[3]	*T*_h_ + *T*_ECC_	6*T*_h_+*T*_CTR_ + 3 *T*_h_ +2 *T*_CTR_	8.22	576	0.0543
[41]	8*T*_h_ + 3*T*_ECC_ + 2*T*_N_	4*T*_h_ + 2*T*_ECC_ + 2*T*_N_ + 3*T*_h_ + 2*T*_ECC_ + 2*T*_N_	31.58	1792	0.2084
[15]	3*T*_h_ + *T*_Kpr_	7*T*_h_ + 2*T*_ECC_ + 2 *T*_Kpr_	9.9	1696	0.0653
Proposed work for ADM	2*T*_h_+*T*_ECC_	3*T*_h_ + 3 *T*_CTR_ +*T*_Kpr_	5.42	800	0.0357

*T_ED_ and *T*_N_ are symmetrical encryption/decryption time, whose values are 5.4 ms and 2.58 ms, respectively.

Table 6 shows that our scheme surpasses existing security schemes in computational and communication efficiency. The proposed scheme is designed in a way that guarantees the lowest number of hashes, CTR, Kyber, and ECC cryptographic operations when compared with other schemes. Furthermore, our scheme yields low latency, and hence achieves high throughput during message exchanges. Notable, the proposed scheme records slightly lower values than other related works, but effectively mitigates the limitations suffered by previous works in handling various cyber and quantum attacks. It also provides robust mutual authentication, as well as forward and backward key secrecy preservation.

#### Energy consumption

In this section, the power consumed by our scheme during the login, authentication and key exchanging processes is estimated. To accomplish this, we use the energy equation that follows.

Powerconsumption=supplyvoltage×current×executiontime(insecond)

where power is measured in mJ. The supply voltage is 3 V, and the current equals 2.2 mA [79]. On the other hand, the execution time is derived as in Table 6.

Consequently, applying the energy equation, the total energy consumed by our scheme is 0.035772 mJ, which is the lowest. As such, our scheme is the best for deployment in the sensor devices, which have limitations in terms of energy.

### Security analysis

In this section, we present both formal and informal proofs to demonstrate the robustness of the proposed hybrid scheme. The sub-sections that follow vividly describes these security analyses in great detail.

#### Informal proof

In this sub-section, we demonstrate that the proposed scheme offers the security features described below.

Proposition 1 *The proposed scheme provides mutual authentication.*

**Proof**: In our scheme, mutual authentication is performed by both parties communicating with each other. This is achieved using private and public keys based on ECC and KEM. As such, only the trusted user can handle encryption, message transfer, and decryption because he/she owns the keys. In addition, verification code *VC* between two parties is crucial during this mutual authentication process. To initiate authentication, the *ADM_i_* sends authentication request *ADM_reqi_ =* {*AN_i_, TR_ADMi_ , RHWP_ADMi_*} to *CHSP*, where *RHWP_ADMi_ = HWP_i_* ⊕ R_1_ and *TR_ADMi_=Enc_sk_* (*T_1_,R_1_*)*.* After receiving this login request, *CHSP* verifies it as follows: Both *T_1_* and *R_1_* are retrieved through decryption as *Dec_ski_* (*TR_ADMi_*). Afterwards, timestamp *T_1_* is verified. Next, login information is recomputed by *XORing* and hashing (*HWP_i_* and *HA_i_*). This is followed by checking their availability in *BC* and IPFS nodes. To retrieve *ADM_iinfo_* decryption is performed using *PrH*. It then creates $HA_{i}^{\prime }$*=h*($AN_{i}^{\prime }∥HPW_{i}^{\prime }$) and validates $HA_{i}^{\prime }$ against *HA_i_* . Provided that these two values are similar, the *ADM* is successfully authenticated. To authenticate CHSP, the Verification Code (*VC*) and *h* (*R*_1_) are encrypted with *SK_i_* and sent to *ADM_i_*. At the *ADM_i_* ,decryption using *Dec_Sk_* is deployed to recover {*VC,h*(*R_1_*)}*.* Next*,* value of *h*(*R_1_*) is used by *ADM_i_* to authenticate the *CHSP*.

Proposition 2 *The proposed scheme preserves user anonymity.*

**Proof.** The proposed system supports anonymity for participated entities. For instance, in the registration phase, the *ADM_i_* sends personal information after being hashed and encrypted with *Pu_H_* as *ADM_iinfo_= Enc_PuH_* (*AN_i_* ∥*HPW_i_* ∥*PH_i_*) so that it is anonymous. In the authentication phase, the login information is also hashed and encrypted before being sent to *CHSP* as *TR_ADMi_=Enc_sk_(T_1_,R_1_), RHWP_ADMi_ = HWP_i_ ⨁ R_1_* and *ADM_reqi_ =* {*AN_i_ , TR_ADMi_ , RHWP_ADMi_*}*.*

Proposition 3 *The proposed scheme maintains confidentiality.*

**Proof**. The data confidentiality is achieved in two ways: The attacker's advantage of compromising confidentiality depends on obtaining the secret key *SK*. However, this key is based on the strength of the KEM algorithm (on the *ADM* side) and ECC algorithm (on the doctor's side). In addition, data confidentiality is upheld by storing and transmitting it in encrypted format. Further protection is assured using *BC* and IPFS technology.

Proposition 4 *The proposed scheme provides privacy.*

**Proof**. In the proposed scheme, patient information privacy is ensured by using *BC*, which gives the patients unique hash codes {*HA_i_*} that can only be recognized by authorized entities. This is facilitated by IPFS, in which data is encrypted, and given distinctive hash {*DH_Pi_*} that is then stored in *BC*. Other system entities are protected using the same mechanisms.

Proposition 5 *The proposed scheme preserves integrity*

**Proof**. Data integrity is preserved through *BC* and SHA256, which store data based on hashing. In so doing, any minor changes can be detected and tracked. For example, doctor *Do_i_* information is hashed as *HA_i_=h_SHA256_*(*DN_i_* ∥*HPW_i_*) prior to being stored with other information in IPFS. Afterwards, their hash address *DH_Di_* is stored in *BC* to give it a unique hash related to the values {*HA_i_, DH_Di_*}*.*In our scheme, data modification is restricted based on the RBAC, which determines which authenticated entity should be granted authorization to access data.

Proposition 6 *The proposed scheme guarantiees forward and backward secrecy.*

**Proof**. Forward and backward secrecy is crucial for keeping past and future communications' confidentiality intact even if a key is compromised. To preserve these properties, we use a single ephemeral session key *SK_ti_* for each session. This key is derived from a combination of time and random numbers, and is used to encrypting and decrypting the communication session. In addition, we employ secure channels for exchanging the keys using ECC and KEM.

Proposition 7 *Our scheme ensures unlinkability.*

**Proof**. This requirement is achieved by using different random nonces of *R_1_* $\in Z_{n}^{*}$ for different sessions. For each login attempt by doctor *Do_i_*, timestamp *T_1_* and *VC* are used to derive *TR_Doi_=Enc_sk_* (*T_1_, R_1_*) and *RHWP_Doi_ = HWP_i_ ⨁ R_1_*, where *TR_Doi_* and *RHWP_Doi_* are temporary and changeable values for each login process. Consequently, an adversary is unable to link different login operations with the same information such as previous, current, and next sessions together.

Apart from the above security features, many security factors were added to the proposed system to enhance its resilience against cyber threats. For instance, session keys provide significant security benefits in various scenarios. This is particularly because they maintain forward and backward secrecy and protect against replay, phishing, quantum, and 51 % attacks. In the propositions below, we show that our scheme resists various attacks.

Proposition 8 *The proposed scheme can resist replay attacks.*

**Proof**. In interactions between *ADMi* and *CHSP*, each session has a unique session key and timestamps that changes upon session completion. Thus, even if an attacker intercepts keys or messages from one session, they cannot use them in future sessions.

Proposition 9 *The proposed scheme is resistance phishing attack.*

**Proof.** The authentication information is dynamic and associated with each particular session. As such, even if a user unknowingly submits his information to a phishing site, the attacker will only have access during that specific session. Since session keys are short-term and specific to each session, any subsequent attempts to use that information require the new session key, which the attacker will not have. For instance, the authentication information for the *ADM_i_* in each session is associated with two variables (that is, random nonce *R_1_* and timestamp *T_1_*) that are changed at the end of each authentication session. In addition, the secret session key *SK_it_* is generated instantaneously and is different for each session. Particularly, the *CHSP* generates *S* (a large random prime number) and timestamp *T_i_*. Thereafter, the shared secret key *SK_it_* is computed as *SK_it_= h*(*S*∥*T_i,_*∥*SK_i_*)*.* This key is also associated with the shared secret key stored in the *ADM*_i_’s registry.

Proposition 10 *The proposed scheme is effective against 51 % attack.*

**Proof**. Although session keys cannot entirely prevent a 51 % attack, they can limit the impact by making it difficult for an attacker to manipulate transactions consistently. With unique session keys for different transactions or sessions, even if an attacker controls the majority of mining resources, they would still face challenges in replaying or successfully carrying out their malicious intentions across multiple sessions or transactions. In our scheme, we deploy cryptographic techniques that are effective in session key management. For instance, we utilize KEM technique for key management, which enhance blockchain integrity.

Proposition 11 *The proposed system mitigates MITM, DoS, and impersonation attacks.*

**Proof**. We deploy different mitigation strategies to mitigate these threats. For instance, we generate a one-time session key *SK_it_* for each session such that even if attackers access the information of any session, they cannot leverage the exchanged parameters between the entities and *CHSP*. Suppose that attacker is interested in gaining values {*AN_i_, HPW_ADM_*} and uses them to log in. However, the adversary lacks values {*R_1_, T_1_*} which are required to successfully authenticate *ADM_i_*. Therefore, the derivation of *TR_ADMi_=Enc_sk_* (*T_1_, R_1_*)*, RHWP_ADMi_ = HWP_i_ ⨁ R_1_, ADM_reqi_ =* {*AN_i_ , TR_ADMi_ , RHWP_ADMi_*} flops*.* In addition, these values are changeable and generated randomly for each login session. Suppose that the adversary has launched MITM attack with the goal of impersonating an IPFS node or *BC* so as to steal patient data. This attack will fail since the information is encrypted before being stored.

Proposition 12 *The proposed scheme withstands quantum attacks.*

**Proof**. In the proposed scheme, quantum attacks are repelled by employing the post-quantum Kyber algorithm. This algorithm generates a strong code that is immune to quantum devices and provides protection for the private key distribution between the *ADM_i_* and the *CHSP* as follows: the *CHSP* generates the shared secret key *SK_it_= h*(*S*∥*T_i,_*∥*SK_i_* ), where *S* and *T*_i_ vary for each session. Next, this derived *SK_it_* is encapsulated with Kyber and *Pu_ADMi_.* In addition, *ADM_i_* retrieves the Cipher-KEY through the decapsulation process by using private key *Pr_ADMi_.*

Proposition 13 *The proposed system faces a Sybil attack.*

**Proof**: The attack is resisted through several strategies. For instance, upon logging in at *ADM*_i_ node, the login parameters {$AN_{i},\;HPW_{ADM}\}$ are computed. Thereafter, values *R*_1_ and *T*_1_ are incorporated in $TR_{ADMi\;}=\;Enc_{SK}\left(T_{1},R_{1}\right)$, and $RHWP{\;}_{ADMi}=\;HWP_{i}\;⊕\;R_{1}$ to form the login request $ADM_{reqi\;}=\;\left\{AN_{i},\;TR_{ADMi},\;RHWP_{ADMi}\right\}$ which is sent to *CHSP*. Before granting access to the network, this login request is checked at the *CHSP* so as to verify the node's identity. On the other hand, *BC* and IPFS technologies are deployed to maintain an immutable record of device identities. This is facilitated by the unique identifier {$HA_{i}$}, which is used to verify device identities prior to communicating in the IoHT.

Proposition 14 *The scheme is capable of defending against hijacking attacks.*

**Proof:** The mutual authentication of the proposed scheme is accomplished through two transactions: *ADM_reqi_ =* {*AN_i_ , TR_ADMi_ , RHPW_ADMi_*} and sending the shared key-encrypted verification code between *ADM* and *CHSP* (that is, *CHSP → ADM_i_ :* {*VC , h* (*R1*)}*_SK_*. These procedures preserve legitimacy of the transmitted messages. In addition, the shared key *SK*_i_ between the authorized *ADM* and *CHSP* ensures exclusive access and decryption capabilities.

Proposition 15 *Our scheme prevents brute-force and dictionary attacks*

**Proof:** These attacks target the strength of the encryption algorithm and the complexity of the keys used, rather than the session management itself. To defend against these attacks, properly implemented keys with sufficient length and complexity are essential. However, a single session key alone cannot prevent these attacks. In the proposed scheme, the key size is 256-bit in length. In addition, the cryptographic algorithms CTR, KEM, and ECC are proven strong, and hence can mitigate brute-force and dictionary attacks.

#### Formal proof

In this section, BAN Logic is employed to demonstrate the robustness of the authentication procedures of the proposed protocol. Specifically, BAN logic is utilized to reveal the robustness of the authentication procedures between *ADM* and *CHSP*. In this proof, we let *A* and *B* represent statements, while *S* and *T* denote the subjects. The notations used during the BAN logic proofs are detailed below.

#(*B*): Message *B* is fresh
*S*|≡ *A*: Subject *S* considers statement *A* to be true
*S*|∼ *A*: At some point, subject *S* sent message *A*
*S*| ◃ A: *S* has seen message *A*
*S*| ⇒*A: S* has control over *A*
*S* $\overset{\;K\;}{↔}$ *T*: Subject *S* and *T* share key *k*
$S_{\;⇌}^{\;K\;}T$ : *k* is the shared secret between principles *S* and *T*
${\left\{A\right\}}_{k}$ : Message *A* is encrypted using key *k*
$\langle A_{k}\rangle$ : Secret *k* is combined with *A*

In addition to the above notations, the following BAN logic rules are deployed.

*R*_1_) Message-meaning rule (*MMR*): $\frac{S|\equiv S\overset{K}{↔}T,S|◃{\left\{A\right\}}_{k}}{S|\equiv T|\equiv ∼A}$
*R*_2_) Nonce verification rule (*NVR*): $\frac{S|\equiv \#(A),S|\equiv T|∼A\;}{S|\equiv T|\equiv A}$
*R*_3_) Jurisdiction rule (*JR*): $\frac{S|\equiv T\Rightarrow A\;,S|\equiv T|\equiv A\;}{S|\equiv A}$
*R*_4_) Believe rule (*BR*): $\frac{S|\equiv (A,B)\;}{S|\equiv A}$
*R*_5_) Freshness rule (*FR*): $\frac{S|\equiv \#(A)\;}{S|\equiv \#(A,B)}$

To demonstrate that our scheme achieves perfect and protected joint validation between ADM and CHSP, the following security goals must be attained.

(1) *ADM* |≡ *ADM* $\overset{\;SK\;}{↔}$ *CHSP*
(2) *ADM* |≡ *CHSP* |≡ *ADM* $\overset{\;SK\;}{↔}$ *CHSP*
(3) *CHSP* |≡ *ADM* $\overset{\;SK\;}{↔}$ *CHSP*
(4) *CHSP* |≡ *ADM* |≡ *ADM* $\overset{\;SK\;}{↔}$ *CHSP*

During the authentication process, three messages are exchanged between *ADM* and *CHSP*. For effective proofs, these messages are converted into idealized format as detailed below:

M_1_) ADM →CHSP : {ADM_req_}_SK_
Idealized form: (ADM $\overset{\;}{↔}$CHSP, ADM_SK_} _ADM_ $\overset{\;SK\;}{↔}$_CHSP_
M_2_) CHSP → ADM_i_ : {VC , h (R_1_)}_SK_
Idealized form: (CHSP $\overset{\;}{↔}ADM\;$, VC_req_} _ADM_ $\overset{\;SK\;}{↔}$_CHSP_
M_3_) ADM_i_ → CHSP : V_res_
Idealized form: (ADM $\overset{\;}{↔}$ CHSP , VR_res_} _ADM_ $\overset{\;SK\;}{↔}$_CHSP_

Next, the following Preliminary State Assumptions (*PSA*_i_) are made regarding our proposed scheme. The freshness and legitimacy of the values in these assumptions are deployed to demonstrate the robustness of the authentication procedures and the session key setup between *ADM* and *CHSP*:

(1) *CHSP* |≡ #*T*
(2) *CHSP* |≡ #*VC*
(3) *ADM* |≡ *ADM* $\overset{\;SK\;}{↔}$ *CHSP*
(4) *CHSP* |≡ *ADM* $\overset{\;SK\;}{↔}$ *CHSP*
(5) *ADM* |≡ C*HSP* |⇒*ADM* $\overset{\;SK\;}{↔}$ *CHSP*

Using these notation, rules, initial state assumptions and idealized messages, we execute the rigorous BAN logic proofs to demonstrate the existence of robust mutual, and secured authentication between *ADM* and *CHSP*.

Based on message *M*_1_, we obtain the following proof:

1. *CHSP ◁ (ADM_SK_) _ADM_* $\overset{SK}{↔}$ *_CHSP_*

From (*PSA*_4_) we get:

2. *CHSP |≡ ADM* $\overset{\;SK\;}{↔}$ *CHSP, ADM_SK_*
The goal 3. Is achieved.
Getting fresh values *T* and *VC* yields:
3. *CHSP |≡ # (T,VC, ADM_SK_, ADM* $\overset{SK}{↔}$ *CHSP)*

Applying *NVR* rule, we get the following.

$$\frac{CHSP\;\left|\equiv \;\#\;\left(T,VC,ADM_{\text{SK}},\;ADM\;\overset{\;SK\;}{↔}\;CHSP\right)\;,\;CHSP\;\right|\equiv \;ADM\;\overset{\;SK\;}{↔}\;CHSP,\;ADM_{\text{SK}}}{CHSP\;\left|\equiv \;ADM\right|\equiv \left(ADM_{\text{SK}},\;ADM\;\overset{\;SK\;}{↔}\;CHSP\right)}$$

This achieves Goal (4).

According to *M*_2_, we get the following:

4. ADM ◁ (VC_req_) _ADM_ $\overset{\;SK\;}{↔}$_CHSP_

Based on *PSA*_3_, we obtain the following.

5. ADM |≡ CHSP |∼(ADM $\overset{\;SK\;}{↔}$ CHSP)

On the other hand, the *FR* rule and *M*_3_ produce the following.

6. ADM |≡ # (T,VC) _ADM_ $\overset{\;SK\;}{↔}$_CHSP_

Using the FR and NVR for *5.* and *6.* yields:

$$\frac{ADM\;|\equiv \;\#\;\left(T,VC\right)ADM\;\overset{\;SK\;}{↔}\;CHSP),\;ADM\;\left|\equiv \;CHSP\right|∼\left(ADM\;\overset{\;SK\;}{↔}\;CHSP\right)}{ADM\;\left|\equiv \;CHSP\right|\equiv \left(\;ADM\;\overset{\;SK\;}{↔}\;CHSP\right)}$$

Therefore, Goal (2) is attained.

7. From *PSA*_5_ and Goal (2) by applying Jurisdiction rule (*JR*):
  $$\frac{ADM\;\left|\;\equiv \;\text{CHSP}\right|\;\Rightarrow ADM\overset{SK}{↔}\;CHSP,\;ADM\;\left|\equiv \;CHSP\right|\equiv \left(\;ADM\;\overset{\;\;\;\;\;\;SK\;\;\;\;\;\;}{↔}\;CHSP\right)\;\;}{ADM|\equiv \left(\;ADM\;\overset{\;\;\;\;\;\;SK\;\;\;\;\;\;}{↔}\;CHSP\right)}$$
The Goal (1) is achieved.

The above analysis demonstrates that the proposed scheme achieves mutual authentication.

#### Formal security analysis using Scyther tool

**Fig. 14:**
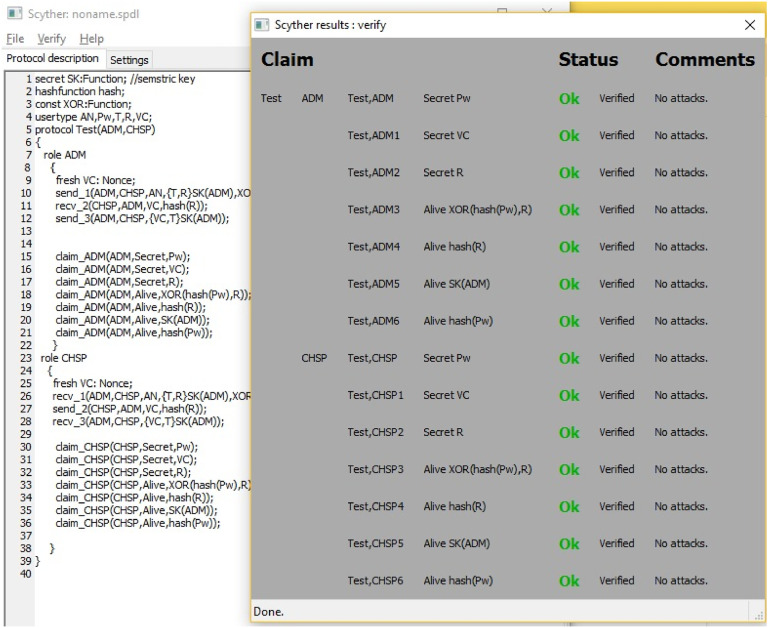
Scyther result.

The Scyther tool is utilized for formal analysis under the perfect cryptography assumption. It was used to identify the security requirements and vulnerabilities of the proposed protocol. To facilitate this analysis, the proposed scheme is encoded according to the Security Protocol Description Language (SPDL), which describes the roles of the users with sequences of events. Fig. 14 interprets the verification result acquired from the proposed scheme under the Scyther tool. It also explains the satisfaction of all security requirements and no attack threats succeeded.

## Conclusion

In this paper, we have developed a post-quantum authentication scheme for IoHT systems utilizing Kyber-512, ECC, CTR, BC IPFS, and fog computing. The proposed scheme has been formally and informally analyzed from the security perspective. BAN logic and Scyther tools were utilized for formal security proof and analysis. The results of these analyses have shown that our scheme is resistant to various cyber threats, such as quantum attacks, phishing, MITM, DoS, impersonation, and 51 % attacks among others. Moreover, our scheme preserved security requirements such as anonymity, confidentiality, privacy, integrity, and unlinkability. From the performance perspective, its computational and communications costs were lower compared with several other related schemes. As such, the proposed scheme is efficient and hence greatly reduces processing time and message costs.

## Limitations

Although the results of the comparison and analysis proved that the proposed scheme is significantly superior in terms of costs (computational, communication, and energy) and security, it still needs practical implementation, which may face compliance and applicability issues with other networks.

## Ethics statements

The submitted paper represents the entire research efforts and analyses of the researchers and accurately and faithfully reflects the comprehensive coverage of the topic.

## CRediT authorship contribution statement

**Enas W. Abood:** Conceptualization, Methodology, Software, Writing – original draft, Writing – review & editing. **Ali A. Yassin:** Conceptualization, Methodology, Software, Validation. **Zaid Ameen Abduljabbar:** Conceptualization, Methodology, Software, Writing – original draft, Writing – review & editing. **Vincent Omollo Nyangaresi:** Writing – original draft, Writing – review & editing. **Ali Hasan Ali:** Validation.

## Declaration of competing interest

The authors declare that they have no known competing financial interests or personal relationships that could have appeared to influence the work reported in this paper.

## Data Availability

No data was used for the research described in the article.
